# Adverse Childhood Experiences and Prescription Opioid Use During Pregnancy: An Analysis of the North and South Dakota PRAMS, 2019–2020

**DOI:** 10.21203/rs.3.rs-2547252/v1

**Published:** 2023-05-09

**Authors:** Alexander Testa, Benjamin Jacobs, Lixia Zhang, Dylan Jackson, Kyle Ganson, Jason Nagata

**Affiliations:** University of Texas at Health Science Center at Houston; Texas Christian University; University of Louisville; Johns Hopkins University; University of Toronto; University of California, San Francisco

**Keywords:** Pregnancy, Adverse Childhood Experiences, Prescription Opioid, PRAMS

## Abstract

**Objectives:**

This study assesses the association between adverse childhood experiences (ACEs) and prescription opioid use during pregnancy.

**Methods:**

This study uses data on 2,999 individuals from the 2019 and 2020 Pregnancy Risk Assessment Monitoring System (PRAMS) from North Dakota and South Dakota. The relationship between ACEs and prescription opioid use during pregnancy is examined using multiple logistic regression.

**Results:**

The prevalence of prescription opioid use increases alongside accumulating ACEs. Compared to those with no ACEs, recent mothers with three or more ACEs have a 2.4 greater odds of prescription opioid use during pregnancy (aOR [adjusted odds ratio] = 2.437; 95% CI [confidence interval] = 1.319, 4.503).

**Conclusion:**

Accumulating ACEs are associated with an increased risk of prescription opioid use during pregnancy. Additional research is needed better understand the mechanisms that link ACEs and prescription opioid use during pregnancy, as well as how to best support those with ACEs exposure in a trauma-informed manner to reduce the risk of substance use.

## Introduction

From 2020 to 2021, over 107,000 drug overdoses occurred in the United States.^1^ A substantial driver of overdose mortality is opioids, which accounted for approximately 76% of all drug overdose deaths in 2021.^1^ The opioid epidemic has touched many segments of the population over the past two decades. However, during the opioid epidemic crisis, pregnant women are a particularly vulnerable population since opioid use not only impacts their health but also poses a risk to fetal health.^2–5^

A growing body of empirical studies confirms a link between exposure to prenatal opioids and poor birth outcomes such as stillbirth, preterm birth, low-birth weight, congenital disabilities, and neonatal abstinence syndrome. ^2,3,6–8^ In addition, prenatal opioid use is associated with long-term health problems, including poor infant motor skills and communication skills,^9^ attention-deficit hyperactivity disorder (ADHD) during school age,^10^ impaired cognitive functioning,^11–14^ and vulnerability to future substance use disorders.^15,16^

Despite the risk opioids pose for maternal and infant health, estimates suggest that nearly seven percent of women have reported using a prescription opioid during pregnancy.^17^ Given the potential harms of prenatal opioid exposure to offspring’s health and development, it is essential to identify the factors associated with prenatal opioid use to inform policy and practice better. Of notable importance is the role of earlier stressful life events in contributing to the risk of opioid use during pregnancy. For instance, research finds that adverse childhood experiences (ACEs)― experiences with abuse, neglect, and household dysfunction during childhood and adolescence―are associated with opioid use.^7–10^ However, limited research has investigated this relationship during pregnancy. One study on this topic uses an unrepresentative sample of 303 pregnant women in a psychosocial perinatal support program in a Southern urban medical clinic, finding no association between accumulating ACEs exposure and opioid use during pregnancy.^18^

Using data on representative samples of live births in two U.S. states, the current study extends prior literature by examining whether accumulating ACEs are associated with a woman’s prescription opioid use during pregnancy.

## Data

Data are from the Pregnancy Risk Assessment Monitoring System (PRAMS). The PRAMS is an ongoing population surveillance system of live births in the United States conducted by the Centers for Disease Control and Prevention (CDC) and state health departments. Data are collected yearly via a stratified systematic sample of birth certificate records. The PRAMS data are from three separate sources: (1) birth certificate records, (2) vital record systems, and (3) responses to a PRAMS survey. The PRAMS survey is mailed to the home address of recent mothers approximately 2 to 4 months following their birth delivery. After up to three mailing attempts, telephone calls are made to non-responders. Survey weights enable adjustment for non-response and non-coverage, thereby making samples representative of live births in a given state.^19^

While the entire PRAMS collects data from 46 states representing approximately 81% of all U.S. live births, a subset of states in specific years ask topic-specific questions. In 2019 and 2020, a supplemental survey was administered to a subset of jurisdictions asking about prescription opioid use during pregnancy.^17^ In addition, select states include topic-specific questions asking mothers about various life experiences. Only two states—North Dakota and South Dakota—have questions asking about mothers’ adverse childhood experiences.^20^ Accordingly, the current study uses data on 2,999 mothers from the 2019 and 2020 PRAMS surveys conducted in North and South Dakota. Appendix A provides a flow chart describing the analytic sample section.

The study was performed in accordance with the Declaration of Helsinki. The study was approved by the Centers for Disease Control and Prevention in accordance with the data usage agreement for the Pregnancy Risk Assessment Monitoring System. All participants provided informed consent; for minors younger than 18 informed consent was waived by CDC. The general PRAMS methodology and protocol have been reviewed and approved by the CDC institutional review board, and state PRAMS projects undergo review by the local institutional review board of record for the health department. An informed consent document in each survey packet explains a participant’s rights for mail surveys. No written consent is required; consent is implied if the survey is completed and returned.^19^ For phone interviews, the informed consent document is read verbally, and the participant verbally agrees to proceed with the survey. Minors younger than 18 years who have given birth are considered emancipated for decisions about their children and do not require consent from parents or guardians to participate.^19^ The PRAMS survey is administered in both English and Spanish. While an interviewer verbally administers phone- based surveys, mail surveys may depend on a women’s literacy levels and comfort in responding to the survey, neither of which are assessed in the PRAMS data collection process, and therefore consent is not requested from a parent or legal guardian in cases when an individual has low literacy levels.^21^

### Dependent Variable

Consistent with prior research,^17,22^ any prescription opioid use is a dichotomous indicator of whether a respondent reported using any prescription opioids during their most recent pregnancy. Respondents were asked, “During your most recent pregnancy, did you use any of the following prescription pain relievers?”: (a) hydrocodone (like Vicodin^®^, Norco^®^, or Lortab^®^), (b) codeine (like Tylenol^®^ #3 or #4, not regular Tylenol^®^), (c) oxycodone (like Percocet^®^, Percodan^®^, OxyContin^®^, or Ultracet^®^), (d) tramadol (like Ultram^®^ or Ultracet^®^), (e) hydromorphone or morpheridine (like Demorol^®^, Exalgo^®^, or Dilaudid^®^), (f) oxymorphone (like Opana^®^), (g) morphine (like MS Contin^®^, Avinza^®^ or Kadian^®^), or (h) fentanyl (like Duragesic^®^, Fentora^®^, or Actiq^®^). Respondents who answered affirmatively to using any of these prescription opioids during pregnancy were coded as a value of 1; those who did not indicate any use of these prescription opioids were coded as 0.

### Independent Variable

ACEs were measured using respondent self-report on ten types of childhood adversity before age 18. The ten questions used to classify ACEs closely approximate the measures from the CDC-Kaiser ACE Study.^23^ Appendix B presents the definitions and prevalence for the ten items. Consistent with prior research using PRAMS data, responses to the 10 ACE items are combined to create a cumulative score ranging from 0–10. The total ACEs scores were grouped into four categories: 0 ACE, 1 ACE, 2 ACEs, 3 or more ACEs.^20,22^

### Control Variables

Control variables include the mother’s age (<18, 18–24, 25–29, 30–34, and 35 or older), mother’s race/ethnicity (White, Hispanic, Black, Native American, Asian/Other and mixed race), mother’s educational attainment (0 = less than college, 1 = college graduate), marital status (0 = not currently married, 1 = currently married), number of prior births (0, 1, 2, or 3+), whether a mother reported being on Medicaid in the three months before pregnancy (1 = yes; 0 = no), household income (≤$16,000, $16,000-$40,000, $40,001-$85,000, or >$85,000), state of residence, and year of birth.

### Analytic Approach

The bivariate association between the number of ACEs and prescription opioid use during pregnancy is assessed using a chi-square (χ^2^) test. Multivariable logistic regression is used to examine the associations between different ACEs categories and prescription opioid use during pregnancy, net control variables. All data analyses were conducted using the svy package for weighted survey data in Stata/S.E. version 17. Variance inflation factors were under 2, indicating no significant issues with multicollinearity.^24^

## Results

**Figure 1:**
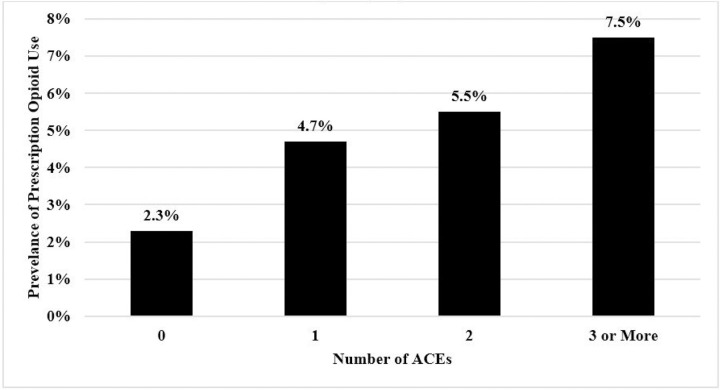
Prevalence of Prescription Opioid Use During Pregnancy by Number of ACEs (*N* = 2,999) *Note:* rests of a χ^2^ with 3 degrees of freedom shows a statistically significant difference between prescription opioid use during pregnancy across the number of ACEs (χ^2^ = 37.69, p <.001)

Summary statistics are presented in Table 1. Overall, 4.7% of the sample reported prescription opioid use during pregnancy; 39.5% reported no ACEs, and 30% of the sample reported three or more ACEs. Figure 1 shows that the prevalence of prescription opioid use increased alongside higher ACEs score: 0 ACEs (2.3%), 1 ACE (4.7%), 2 ACEs (5.5%), and 3 or more ACEs (7.5%). A chi-square test reveals a statistically significant difference between the prevalence of prescription opioid use in different ACEs groups (χ^2^ = 37.69, *p* < .001).

The results of the multivariable logistic regression in Table 2 shows that compared to those with no ACEs, recent mothers with three or more ACEs had approximately a 2.4 greater odds of prescription opioid use during pregnancy (aOR [adjusted odds ratio] = 2.437; 95% CI [confidence interval] = 1.319, 4.503). The multiple logistic regression analysis Appendix B detail that eight of the 10 ACEs (except for parental separation and household violence) had a positive and statistically significant association with prescription opioid use during pregnancy.

## Discussion

The core findings of this study reveal that accumulating ACEs—especially three or more ACEs—were associated with a 2.4-fold increase in the odds of prescription opioid use during pregnancy. These findings confirm earlier research that found a connection between ACEs and opioid use.^18, 25–27^ However, the results also differ from recent work conducted by Osofsky et al.^18^, which found no association between accumulating ACEs and opioid use during pregnancy among a sample of 303 pregnant women embedded in a psychosocial perinatal support program in a Southern urban medical clinic.

The findings of this study expand upon prior research by offering critical evidence that the relationship between accumulating ACEs and prescription opioid use in adulthood extends to the prenatal period. The finding of elevated patterns of prescription opioid use during pregnancy among women who experienced three or more ACEs offers an essential insight into the enduring role of early life adversity on health behaviors during pregnancy.^28^ It is also important to note, however, that most women—even in the face of ACEs exposure—did not use prescription opioids during pregnancy. For instance, while 7.5% of women with three or more ACEs used opioids during pregnancy, over 90% of respondents with high ACEs exposure did not use opioids during this period. Accordingly, while ACEs increased the risk of prenatal opioid use, this was not the case for most pregnant women, suggesting that high levels of early life adversity increase the risk of, but do not guarantee, compromised behavioral health during pregnancy.

While the current study established a connection between ACEs and prescription opioid use during pregnancy, future research needs to explore potential pathways to explain this relationship. For instance, many recent studies have documented that ACEs are strongly associated with chronic pain in children and adults,^4, 29–32^, which may directly lead to prescription opioid use. Additionally, the trauma stemming from ACEs may result in psychological or behavioral adaptions that are important for short-term survival but may place an individual at long-term risk for engaging in risky health behaviors such as substance use.^28^ Likewise, ACEs can result in challenges with emotional regulation and a propensity for outlets to alleviate adverse emotional states.^33,34^ Given that pregnancy is an emotionally vulnerable period, such states may be heightened during times of pregnancy, thereby amplifying the risk of prescription opioid use. Uncovering the mechanisms of why ACE exposure leads to an increased risk of prescription opioid use during pregnancy for some women is crucial for developing programmatic interventions to provide support to ACEs exposed populations and promote positive health behaviors and healthy pregnancy.

The results also highlight the importance of detecting and mitigating ACEs’ negative repercussions on behavioral health. One means may be using clinical screenings during prenatal care and primary care visits to better detect the presence of ACEs and, when detected, provide trauma-informed care to help ensure a healthy pregnancy. For instance, recent evidence from two pilot studies in the Kaiser-Permanente system^35^ found ACEs screenings can be feasibly conducted in a prenatal care setting without re- traumatization,^36^ and such practices can improve women’s health outcomes and children. Therefore, assessing the feasibility of such approaches to mitigate prescription opioid use during pregnancy is an important area for future research to consider carefully.

## Limitations

There are limitations to the current analysis that can be expanded upon in future research. First, North Dakota and South Dakota were the only two states which asked questions about ACEs and prescription opioid use during pregnancy in the PRAMS study. Accordingly, the results may not be generalizable outside of these contexts, especially considering that these two states are unique in many regards, such as being more rural and having higher populations of White and Native American persons compared to the U.S. general population. Second, the questions about ACEs and prescription opioid use may be subject to recall or social desirability bias. Third, the focus of the study was on a range of prescription opioids used during pregnancy. However, the findings of this study cannot be generalized to the use of illicit non-prescription opioids such as heroin. Fourth, because of sample limitations, we could not examine other questions about rarer outcomes related to opioid use, such as whether opioids were used for pain management or non-medical reasons.^22^ Larger-scale quantitative studies and qualitative research would be helpful to ascertain better the relationship between ACEs and specific reasons for and patterns of opioid use during pregnancy. Finally, because the PRAMS data are cross-sectional, the findings should be interpreted as associations rather than causal relationships.

## Conclusion

ACEs and prescription opioid uses are serious public health concerns that can influence maternal and infant health. The current study offered a novel insight into the relationship between accumulating ACEs and prescription opioid use during pregnancy. The findings suggest the need for additional research to understand better the mechanisms that lead to a link between ACEs and opioid use during pregnancy, as well as how to support those with ACEs exposure in a trauma-informed manner to reduce the risk of subsequent substance use.

## Supplementary Material

Supplement 1

## Figures and Tables

**Table 1:** Weighted Summary Statistics of Analytic Sample (*N* = 2,999)

Variable	%
Prescription Opioid Use	4.7%
*Number of ACEs*	
0	39.5%
1	19.0%
2	11.5%
3 or More	30.0%
*Mother’s Age*	
Less than 18	0.6%
18–24	19.4%
25–29	34.3%
30–34	31.7%
35 or Older	13.9%
*Mother’s Race/Ethnicity*	
White	75.1%
Hispanic	4.8%
Black	4.4%
Native American	9.7%
Asian/Other	2.5%
Mixed Race	3.4%
*Mother’s Educational Attainment*	
Less than High School	9.2%
High School Graduate	20.7%
Some College	30.4%
College Graduate	39.8%
Currently Married	68.5%
*Number of Prior Births*	
0	34.9%
1	31.2%
2	18.8%
3+	15.0%
Medicaid	12.9%
*Household Income*	
≤ $16,000	15.0%
$16,000-$40,000	17.7%
$40,001 – $85,000	36.5%
> $85,000	30.9%
*State of Residence*	
North Dakota	43.4%
South Dakota	56.6%

**Table 2:** Results of Multiple Logistic Regression of Number of ACEs on Prescription Opioid Use During Pregnancy and Covariates (*N* = 2,999)

Number of ACEs	OR	95% CI
0 (Reference)	—	—
1	1.884	(0.976 – 3.638)
2	1.986	(0.903 – 4.368)
3 or More	2.437**	(1.319 – 4.503)

** *p* <.01

Control variables include: mother’s age, mother’s race/ethnicity, mother’s educational attainment, currently married, number of prior births, Medicaid, income, state of residence, and year of birth

*Abbreviations:* OR = odds ratio; CI = confidence interval, ACEs = adverse childhood experiences
