# Supplementary material for: Adverse Childhood Experiences and Prescription Opioid Use During Pregnancy: An Analysis of the North and South Dakota PRAMS, 2019–2020
Source: Res Sq. 2023 May 9:rs.3.rs-2547252. Preprint. [Version 1] doi: 10.21203/rs.3.rs-2547252/v1 (PMC10197742; doi:10.21203/rs.3.rs-2547252/v1)
Supplement: Supplement 1 [file NIHPPRS2547252V1-supplement-1.pdf]

Supplementary Files

This is a list of supplementary files associated with this preprint. Click to download.

- SupplementalMaterial.docx
